# Antimicrobial and Antioxidant Effects of Kappa-Carrageenan Coatings Enriched with Cinnamon Essential Oil in Pork Meat

**DOI:** 10.3390/foods11182885

**Published:** 2022-09-17

**Authors:** Shoukui He, Yifei Wang

**Affiliations:** 1 Department of Food Science & Technology, School of Agriculture and Biology, Shanghai Jiao Tong University, Shanghai 200240, China; 2 Department of Food Science & Technology, School of Perfume and Aroma Technology, Shanghai Institute of Technology, Shanghai 201418, China

**Keywords:** carrageenan, essential oil, edible coating, pork meat, antimicrobial activity, antioxidant effect

## Abstract

Fresh pork is susceptible to microbial contamination and lipid oxidation, which leads to food safety and quality issues. This study aimed to develop a kappa-carrageenan (KC) coating embedded with cinnamon essential oil (CEO) for antimicrobial and antioxidant purposes in pork meat. The uncoated controls and coated samples were subjected to microbial (total viable count, lactic acid bacteria, and H_2_S-producing bacteria), chemical (DPPH and pH), and physical (surface color) analyses during refrigerated storage at 4 °C for 7 days. It was observed that KC coatings exhibited a better preservation effect on pork meat after the addition of CEO. The KC–CEO coatings were effective in retarding the growth of total viable count, lactic acid bacteria, and H_2_S-producing bacteria. In a DPPH test, the level of lipid oxidation in pork meat was also significantly (*p* < 0.05) reduced by the KC–CEO coatings. Furthermore, these coatings displayed pronounced activity in inhibiting the adverse alterations of pH value and surface color. Practically, KC–CEO-coated samples still exhibited an attractive bright red color at the end of refrigerated storage. Taken together, the developed KC–CEO coatings exerted pronounced antimicrobial and antioxidant activities in pork, thus providing a potential approach to preserving perishable meat.

## 1. Introduction

Fresh pork is among the most commonly consumed meat in China and other countries. Its consumption has increased to 55,950,000 tons in 2018, accounting for approximately 64% of total meat consumption in China [1]. However, pork meat is vulnerable to spoilage due to its high protein, fat, and moisture content [2]. Microbial contamination and chemical oxidation are considered key factors responsible for pork deterioration [3]. Microbial deterioration is mainly due to the proliferation of meat spoilage microorganisms such as lactic acid bacteria and H_2_S-producing bacteria [4]. Chemical spoilage can be caused by lipid and protein oxidation in meat [5]. In this context, synthetic additives (e.g., butylated hydroxytoluene and butylated hydroxyanisole) have been utilized for meat preservation for many years [6]. Owing to the adverse health effects caused by these chemicals and the increased consumer demand for food safety, a growing interest has been recently directed towards the exploration of natural antimicrobial compounds such as essential oils (EOs) [7].

Cinnamon essential oil (CEO) stands out for its excellent antimicrobial and antioxidant activities [8,9,10]. The usage of this natural agent is generally recognized as safe by the Food and Drug Administration of the United States [11,12]. Nevertheless, the direct blend of CEO and other EOs into foods faces great challenges due to their hydrophobicity, instability, and intense aroma, which limits their application value in the food industry [13]. In this sense, edible coatings have been proposed as a feasible conveying system that is conducive to promoting the distribution capacity and the antimicrobial activity of EOs in foods [14]. Furthermore, the incorporation of EOs into edible coatings is also able to minimize the effective dose required for in vivo tests, thus reducing the impact of EOs on food organoleptic characteristics [15]. A number of coatings based on chitosan or gelatin were employed to load CEO for the preservation of meat (e.g., roast duck), seafood (e.g., rainbow trout), and fruits (e.g., jujube, mango) [16,17,18,19,20,21,22]. It is thus expected that active packaging coatings based on CEO may have supplementary applications in retarding pork spoilage.

In recent years, much attention has been focused on carrageenan as a source of biopolymer for the formulation of edible coatings [23]. Carrageenan is a natural water-soluble polysaccharide derived from red algae. It is typically divided into three main types, namely, kappa, iota, and lambda. Kappa-carrageenan (KC) is an excellent biopolymer to be used in active food packaging systems considering its high gelling capacity and biocompatibility [24]. Characterization of KC films enriched with EOs of *Zataria multiflora* Boiss, *Mentha pulegium*, and *Satureja hortensis* was carried out in previous studies [25,26,27]. However, there is a lack of knowledge regarding the application of KC films/coatings with EOs as an active agent for meat preservation.

Practically, several KC coatings carrying antimicrobial agents were applied for food preservation. For example, KC coatings loaded with ZnO nanoparticles delayed decay and discoloration, maintained firmness, and reduced the acidity of mango during storage [28]. Furthermore, KC coatings incorporating lemon oil protected trout fillets from microbial growth and lipid oxidation [29]. It is thus expected that carrageenan-EO complex coatings may be promising for preservation applications in the meat industry.

The purpose of the current work was to assess the antimicrobial and antioxidant activities of a KC coating incorporated with CEO in pork meat, which will contribute to knowledge-based utilization of edible coatings during food storage.

## 2. Materials and Methods

### 2.1. Materials

CEO was provided by Jofont Ltd. (Kunshan, China), with E-cinnamaldehyde, eugenol, benzaldehyde, and phenylacetaldehyde as the major components as determined in our previous work [15]. This EO was selected due to its excellent antimicrobial and antioxidant activities [15,30].

Food-grade KC was purchased from Beilian Company (Fengxian, China). Lean pork meat was procured from a local supermarket and transported to our laboratory in sealed foamed polystyrene boxes containing crushed ice within 1 h. All the media and other chemicals were obtained from Sinoreagent Ltd. (Huangpu, China).

### 2.2. Formulation of KC-Based Coatings

KC-based coatings were prepared based on the method of Seol et al. [31] and Wang et al. [15]. Briefly, KC was dissolved in hot water preheated to 90 °C to achieve a concentration of 2% (*w*/*v*). Glycerol was also added at a ratio of 1.5% (*v*/*v*) as a plasticizer. After stirring for 1 h, the mixture was cooled to 60 °C and incorporated with Tween 80 (0.2%, *v*/*v*). CEO was then enriched to the desired level of 0% and 2% (*v*/*v*), respectively, followed by stirring for 30 min and homogenization for 2 min. The resulting solution was utilized for the subsequent pork meat coating test.

### 2.3. Coating Treatment of Pork Meat

A pork coating test was conducted according to He et al. [32]. Pork meat (approximately 10 g each slice) was submitted to a 1 min dip in sterile distilled water (uncoated control), 2% KC coating (KC coating) or 2% KC coating incorporating 2% CEO (KC–CEO coating), respectively. The samples were air-dried in an aseptic laminar-flow condition for 1 h. The uncoated and coated samples were then transferred to polystyrene trays, sealed with polyethylene bags, and stored at 4 °C for 7 days. Pork meat in each group was randomly sampled on days 0, 5, and 7 for microbial (total viable count, lactic acid bacteria, and H_2_S-producing bacteria), chemical (DPPH and pH), and physical (surface color) analyses.

### 2.4. Evaluation of Microbial Property

Ten grams of pork samples in each group were aseptically added into 90 mL sterile saline solution (0.85%, *w*/*v*) in a stomacher bag, followed by homogenization for 2 min at room temperature. Decimal dilutions were then prepared with the same solution. Appropriate dilutions of pork homogenates (100 μL) were spread onto plate count agar and incubated at 28 °C for 2 days to determine total viable count (TVC). Appropriate dilutions (1 mL) were also poured onto MRS (de Man Rogosa Sharpe) agar and allowed to solidify, followed by pouring the same medium over the surface to generate another layer. Lactic acid bacteria (LAB) were enumerated on the double-layered plates of MRS medium after incubation at 25 °C for 3 days. In addition, 1 mL inoculum from the appropriate 10-fold dilution was poured onto Iron Agar and incubated at 30 °C for 3 days for H_2_S-producing bacteria (HSPB) count. All microbiological data were expressed as log CFU/g.

### 2.5. Measurement of Antioxidant Activity

A DPPH assay was utilized to determine the antioxidant activity of KC-based coatings in pork meat [15]. An aliquot (0.1 g) of pork meat was transferred to a glass tube containing 5 mL DPPH (0.004%, *w*/*v*). The samples were constantly mixed at 25 °C for 0.5 h. The absorbance was then taken at 517 nm as an indicator of antioxidant activity. A higher absorbance value corresponded to a lower antioxidant capacity of KC-based coatings.

### 2.6. Measurement of pH

A total of 10 g pork meat was blended with sterile distilled water (90 mL), followed by homogenization at 12,000 rpm for 1 min (Wiggen Hauser, D-500, Berlin, Germany). The resulting homogenate was submitted to pH determination by a PHS-3C digital pH meter (INESA Scientific Instrument Co., Ltd., Shanghai, China).

### 2.7. Determination of Color

A WSC-S colorimeter (Precision Scientific Instrument Co., Ltd., Shanghai, China) was employed to assess the superficial color of pork meat. The parameter of L*, a*, and b* was recorded at different positions of samples. The C* (chroma) and *h*° (hue angle) values were then calculated by the equations as stated below [32]:

C* = (a*^2^ + b*^2^)^0.5^

*h*° = tan^−1^(b*/a*) (a* > 0, b* > 0) or *h*° = 180° + tan^−1^(b*/a*) (a* < 0, b* > 0)

### 2.8. Statistical Analysis

Three replicates of pork meat were prepared for each treatment on each sampling day, and each sample was measured at least in duplicate. The obtained data were compared via the one-way Duncan’s ANOVA test, where *p* < 0.05 was regarded as a significant difference.

## 3. Results and Discussion

### 3.1. Impact of KC-Based Coatings on Microbial Properties of Pork Meat

**Figure 1:**
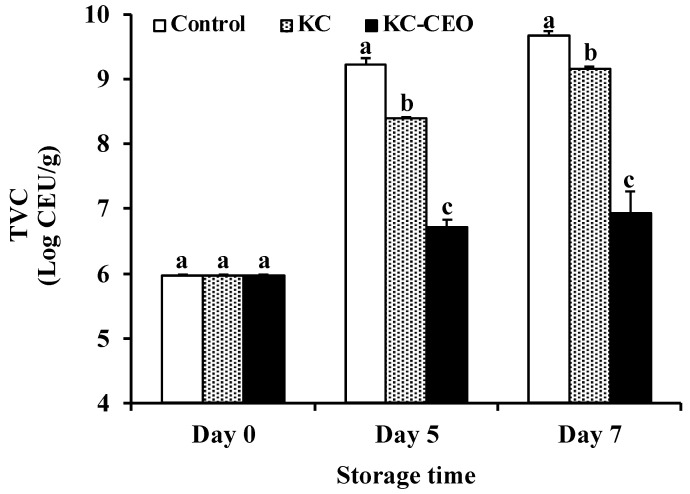
Impact of KC-based coatings on TVC of pork meat during refrigerated storage (4 °C). KC, kappa-carrageenan; CEO, cinnamon essential oil; TVC, total viable count. Different letters for data points on each day represent significant differences (*p* < 0.05).

The proliferation of spoilage microorganisms is a major cause of meat deterioration during storage [3]. Therefore, microbiological indicators (e.g., TVC, LAB, and HSPB) were assessed to determine the antimicrobial activity of KC–CEO coatings in pork meat in the current work. TVC, an important index to assess the quality of pork meat, was greatly affected by the coating treatments during refrigerated storage (Figure 1). The initial TVC for all pork samples was approximately 5.96 log CFU/g, which was close to that reported for sheep and goat meat (5.70 log CFU/g) by Ahmed et al. [33] and that for fresh chicken meat (5.41 log CFU/g) by Zhang et al. [34], respectively. TVC showed a gradual increase in all groups over time, but a lower rate of increase was observed in coated samples compared with uncoated controls. This value began to exceed 8.00 log CFU/g in both uncoated and KC-coated groups after 5 days of storage, while that in the KC–CEO-coated samples was still 6.94 log CFU/g on day 7. According to the TVC threshold (7.00 log CFU/g) recommended for fresh meat [35], KC–CEO coatings were able to delay the spoilage of pork meat for at least 2 days compared to the control. This finding demonstrated the feasibility of incorporating CEO into KC coatings for pork meat preservation.

**Figure 2:**
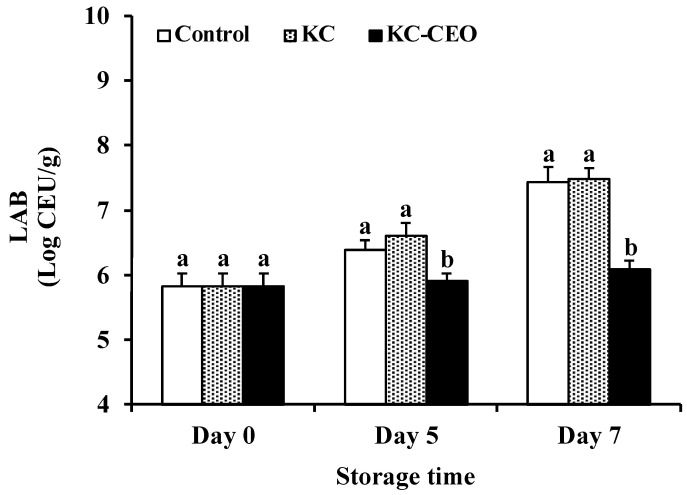
Impact of KC-based coatings on the number of LAB in pork meat during refrigerated storage (4 °C). KC, kappa-carrageenan; CEO, cinnamon essential oil; LAB, lactic acid bacteria. Different letters for data points on each day represent significant differences (*p* < 0.05).

LAB are also critical spoilage microorganisms responsible for the deterioration of food products such as fresh meat and ready-to-eat seafood [36,37]. As shown in Figure 2, the number of LAB in uncoated samples increased significantly (*p* < 0.05) from 5.82 log CFU/g on day 0 to 6.39 and 7.44 log CFU/g on day 5 and day 7, respectively. Moreover, treatment with KC coatings did not significantly (*p* > 0.05) affect the LAB count in pork meat during refrigerated storage, which was in agreement with the observation that KC films did not exert antibacterial activity either via direct contact or by vapor phase [26]. On the contrary, KC–CEO coatings were able to inhibit the growth of LAB in pork meat; LAB count in the KC–CEO-coated samples was 0.48 and 1.35 log CFU/g lower than that in uncoated controls on day 5 and day 7, respectively. Similarly, cinnamon bark oil suppressed the population of LAB in lamb meat by 0.6–1.9 log CFU/g during 16 days of storage at 4 °C [38]. It was indicative that CEO acted as an active agent in KC–CEO coatings, thus exhibiting a good aptitude in inhibiting the growth of LAB in pork meat in the current work.

**Figure 3:**
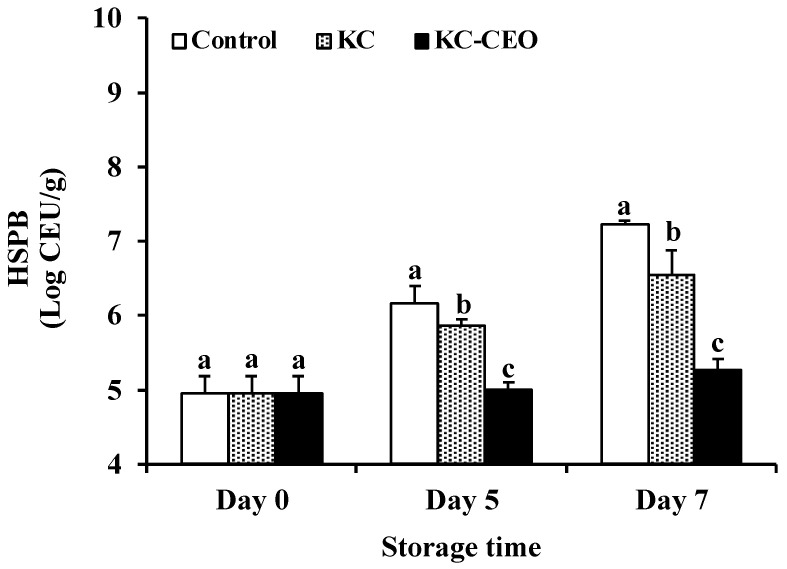
Impact of KC-based coatings on the number of HSPB in pork meat during refrigerated storage (4 °C). KC, kappa-carrageenan; CEO, cinnamon essential oil; HSPB, H_2_S-producing bacteria. Different letters for data points on each day represent significant differences (*p* < 0.05).

The rapid growth of HSPB can also result in the deterioration of food products [39]. As depicted in Figure 3, the initial count of HSPB was approximately 5.00 log CFU/g in all groups. The HSPB count in each group increased dramatically during storage, except for the KC–CEO-coated samples in which the HSPB population remained almost unchanged. On day 7, the lowest value was detected in KC–CEO groups (5.26 log CFU/g), followed by KC samples (6.55 log CFU/g) and uncoated controls (7.23 log CFU/g). These results suggested that KC-based coatings exerted an inhibitory effect on the growth of HSPB in pork meat. Similar results were observed for the anti-HSPB effect of chitosan–caffeic acid coatings on pompano [40], gelatin–eugenol emulsion coatings on Chinese seabass [41], and chitosan–polyphenol coatings on large yellow croaker [42]. Considering that the proliferation of HSPB can result in off-flavors of food products [43], the inhibition of HSPB growth by KC-based coatings in the current work will potentially contribute to improving the sensory quality of pork meat.

Taken together, the application of KC and KC–CEO coatings was able to inhibit the growth of some spoilage microorganisms in pork meat, and the latter one with CEO incorporated showed the most pronounced effect. The additive antimicrobial effect of CEO with KC coatings might be closely related to its pronounced capacity to disrupt bacterial cell membranes and cell walls as revealed in our previous work [30]. Thus, KC–CEO coatings were the most effective in retarding the growth of spoilage microorganisms in pork meat.

### 3.2. Impact of KC-Based Coatings on DPPH Contents of Pork Meat

**Figure 4:**
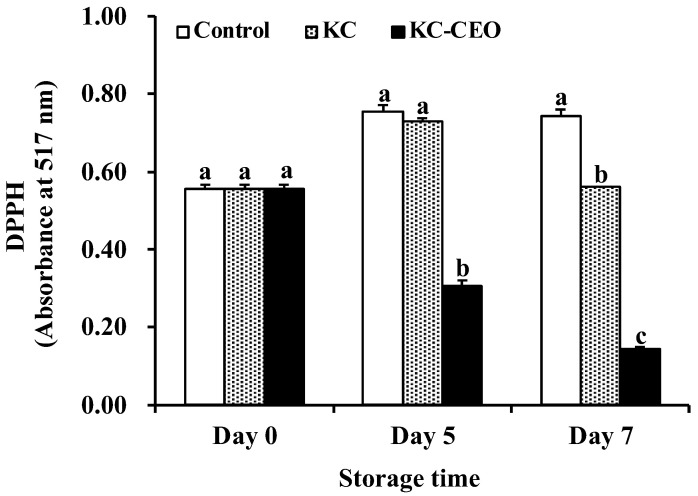
Impact of KC-based coatings on the DPPH scavenging activity of pork meat during refrigerated storage (4 °C). KC, kappa-carrageenan; CEO, cinnamon essential oil. Different letters for data points on each day represent significant differences (*p* < 0.05).

Lipid oxidation is another crucial factor affecting meat quality [3]. Therefore, a DPPH assay was carried out to determine the level of lipid oxidation in pork meat and the results were presented as the absorbance at 517 nm. A lower absorbance value indicated a higher antioxidant activity of edible coatings. As shown in Figure 4, only KC–CEO coatings exerted pronounced antioxidant activity in pork samples after 5 days of refrigerated storage. On day 7, pork samples packaged with both KC and KC–CEO coatings demonstrated a significantly (*p* < 0.05) lower level of lipid oxidation than the uncoated controls. Moreover, the highest antioxidant activity was achieved by the use of KC–CEO coatings, suggesting the feasibility of the incorporation of CEO into KC coatings to enhance the antioxidative effect in pork samples.

In agreement with the aforementioned observations, Shojaee-Aliabadi et al. [26] reported that pure KC films possessed low DPPH-scavenging activity in vitro, probably due to the presence of naturally occurring polyphenols. In addition, the in vitro antioxidant power of KC films was promoted by the addition of *Satureja hortensis* EO. Interestingly, our current work uncovered that KC coatings exerted a modest antioxidant activity in a real food system, which could be due to the barrier action of these coatings between the oxygen and the samples delaying the myoglobin oxidation process in pork meat [44]. Moreover, CEO was able to enhance the antioxidant activity of KC coatings in vivo, which informs current knowledge on food preservation applications of this biodegradable material. The antioxidant ability of CEO was mainly attributed to its phenolic compounds such as phenolic acids, proanthocyanidins, and coumarins [45].

### 3.3. Impact of KC-Based Coatings on pH Values of Pork Meat

**Figure 5:**
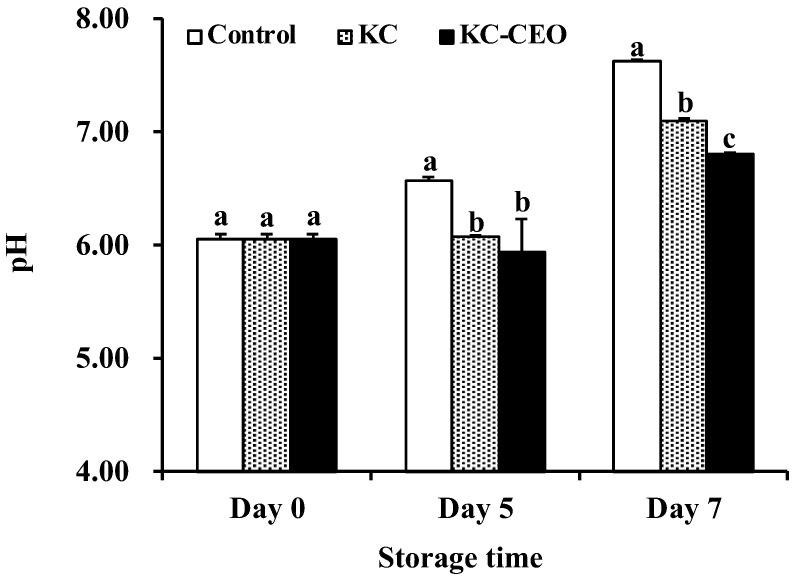
Impact of KC-based coatings on the pH value of pork meat during refrigerated storage (4 °C). KC, kappa-carrageenan; CEO, cinnamon essential oil. Different letters for data points on each day represent significant differences (*p* < 0.05).

The pH value is an important indicator of the quality of fresh meat [15]. Therefore, variations in the pH of pork samples during storage at 4 °C were determined in the current work. As shown in Figure 5, the initial pH of pork meat was 6.05 before the application of edible coatings, which was in agreement with previous reports [46,47]. This value went up markedly in all samples at the end of refrigerated storage, and the increase was more pronounced for the control groups, reaching 7.62 on day 7. The rise in pH over time could be attributed to the accumulation of amines, ammonia, and other alkaline compounds generated by microbial and autolytic reactions [48].

It was noted that pork samples coated with KC and KC–CEO exhibited much lower pH values compared with the uncoated controls (Figure 5), which was related to the capacity of these coatings to disrupt microbial and autolytic reactions. In addition, the KC–CEO coatings demonstrated a better effect than the pure KC coatings for inhibiting the increase in pH on day 7; the final pH of uncoated, KC-coated, and KC–CEO-coated pork was 7.62, 7.09 and 6.80, respectively (Figure 5). This was probably due to the incorporation of the active antimicrobial agent, CEO, into the KC coatings. In fact, CEO contained various antibacterial compounds such as cinnamaldehyde, eugenol, and benzaldehyde [15]. It was thus indicated that the KC–CEO coatings might be more powerful than the pure KC coatings to retard pH increase during pork storage.

### 3.4. Impact of KC-Based Coatings on Color Attributes of Pork Meat

**Figure 6:**
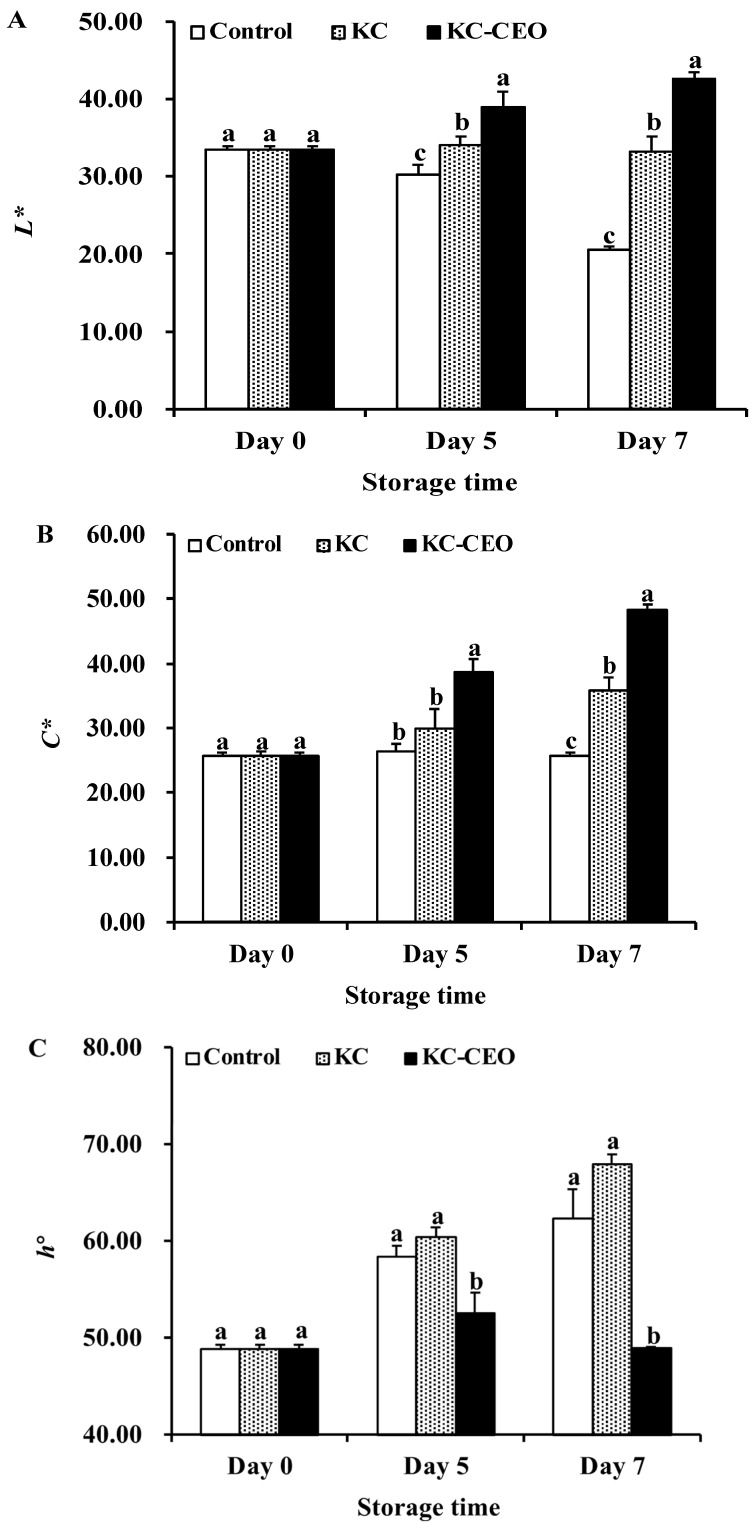
Impact of KC-based coatings on the color parameter of L* (**A**), C* (**B**), and *h*° (**C**) in pork meat during refrigerated storage (4 °C). KC, kappa-carrageenan; CEO, cinnamon essential oil. Different letters for data points on each day represent significant differences (*p* < 0.05).

Color is a key factor in consumer preference for pork meat [32]. Hence, three color attributes (i.e., L*, C*, and *h*°) of pork samples were determined in the current work. The L* value of uncoated, KC-coated, and KC–CEO-coated pork samples decreased, remained unchanged and increased during refrigerated storage, respectively (Figure 6A). All pork samples exhibited increased C* values over time except for the uncoated ones (Figure 6B). Moreover, significantly lower *h*° values were found in the KC–CEO-coated pork samples compared with those in the uncoated and KC-coated groups (Figure 6C).

L* reflects lightness, which ranges from 0 (black) to 100 (white). C* represents color saturation, which varies from dull (low value) to vivid color (high value). *h*° indicates a color wheel, which ranges from red–purple (0°) to yellow (90°) [32]. In the current work, the decreased L* value, remaining C* value, and increased *h*° value of the uncoated samples indicated an unattractive brown and dull color, which might result in a decrease in their consumer acceptability. For KC-coated samples, the increased *h*° value could also lead to discoloration to some extent, despite the remaining L* value and increased C* value. On the contrary, the KC–CEO-coated pork samples exhibited an attractive bright, vivid, red color as suggested by the L*, C*, and *h*° values, respectively. This desirable color stability could be due to the pronounced antimicrobial and antioxidant activities of the KC–CEO coatings in pork meat [32].

Similarly, the incorporation of oregano EO into pectin edible coatings was able to minimize color changes in pork loins [3]. Furthermore, xanthan-gum-based coatings containing EO nanoliposomes could maintain the color stability of salmon [49]. Interestingly, our current work demonstrated that CEO was feasible to be loaded into KC coatings for the maintenance of desirable meat color, which provided an additional option for the selection of powerful biopolymers for food preservation.

## 4. Conclusions

The addition of CEO into KC coatings was able to inhibit microbial growth of TVC, LAB, and HSPB in refrigerated pork meat. Moreover, KC coatings enriched with CEO successfully retarded lipid oxidation and adverse alterations in pH and color throughout storage. Hence, the formulated KC–CEO coatings provide a promising approach for the preservation of perishable meat in the food industry. A systematic sensory analysis of pork meat can be considered in future studies to further demonstrate the strengths of these coatings.

## Data Availability

Data is contained within the article.
